# Benefits of daily online plan adaptation with reduced margins in neoadjuvant chemoradiotherapy for esophageal cancer

**DOI:** 10.1016/j.phro.2026.100946

**Published:** 2026-03-14

**Authors:** Leigh A.P. Bruijs, Thomas Weststrate, Karin N. Goudschaal, Irma W.E.M. van Dijk, Jorrit Visser, Joost J.C. Verhoeff, Zdenko van Kesteren, Tezontl S. Rosario, Arjan Bel, Peter S.N. van Rossum

**Affiliations:** a Amsterdam UMC Location University of Amsterdam, Department of Radiation Oncology, Meibergdreef 9, Amsterdam, the Netherlands; b Amsterdam UMC Location Vrije Universiteit Amsterdam, Department of Radiation Oncology, De Boelelaan 1117, Amsterdam, the Netherlands; c Cancer Center Amsterdam, Treatment and Quality of Life, Amsterdam, the Netherlands

**Keywords:** Esophageal cancer, Radiotherapy, Chemoradiotherapy, Online adaptive, IGRT

## Abstract

**Background and purpose:**

Image-guided radiotherapy (IGRT) for esophageal cancer requires large planning target volume (PTV) margins to account for interfraction variations, increasing radiation-associated side-effects. Cone-beam computed tomography-based (CBCT) online adaptive radiotherapy (oART) enables daily contour and plan adaptation, addressing anatomical changes and allowing for reduced PTV margins while ensuring target coverage. This study compared the dose-volume parameters of daily oART to non-adaptive IGRT in patients with esophageal cancer.

**Material and methods:**

Ten patients with distal esophageal or gastroesophageal junction cancer who received neoadjuvant chemoradiotherapy (41.4 Gy/23 fractions) were included retrospectively. Daily IGRT CBCTs were used to emulate oART with artificial intelligence (AI)-assisted target and organs-of-interest contouring, manual edits when necessary, and adaptive re-optimization. PTV margins (Anterior-Posterior, Left-Right, Caudal-Cranial) were reduced from 5, 7, 10 mm (IGRT) to 3, 5, 5 mm (oART), respectively. Mean organs-of-interest and fraction-equivalent (FE) dose-volume metrics between delivered and adaptive plans were compared.

**Results:**

Compared with IGRT, oART plans significantly reduced the number of fractions with internal clinical target volume underdosing (V_95%_ < 98%: n = 32 vs. n = 1 fraction; p = 0.002) and median hotspot dose ($D_{{0.1cm}^{3}}$: 106.2% vs. 103.9%; *p* = 0.002). Mean heart dose decreased by 10% (*p* = 0.037), and heart FE-V_30Gy_ by 42% (7.3% vs. 4.3%; *p* = 0.002). Mean lung dose was reduced by 11% (*p* = 0.002), lung FE-V_20Gy_ by 39% (4.2% vs. 2.6%; *p* = 0.002), and lung FE-V_10Gy_ by 22% (24.6% vs. 19.3%; *p* = 0.002).

**Conclusion:**

CBCT-based oART improved dose distribution in esophageal cancer by enabling PTV margin reduction, improved target coverage and superior organs-of-interest sparing. These findings encourage clinical implementation to reduce radiation-associated side-effects.

## Highlights

• Emulated adaptive treatment for esophageal cancer was feasible in all 230 fractions.
• Adaptive treatment prevented internal target underdosing in 31/230 fractions.
• Adaptive treatment enabled a median 24.6% reduction in planning target volume.
• Adaptive treatment reduced mean heart and lung dose by 10% and 11%, respectively.

## Graphical abstract

**Figure:**
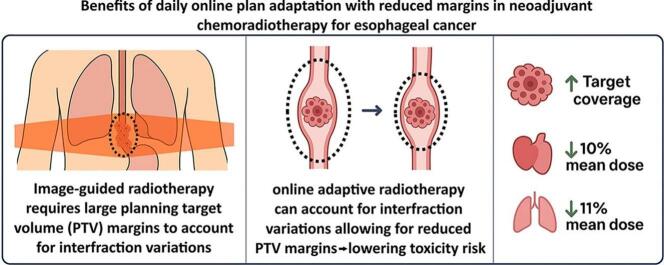

## 1 Introduction

Esophageal cancer is the eleventh most common cancer worldwide (>510.000 new cases/year) [1], and the seventh leading cause of cancer-related mortality (∼450.000 deaths/year) [2]. A standard treatment option for locally advanced esophageal or gastro-esophageal junction (GEJ) carcinoma is neoadjuvant chemoradiotherapy (nCRT) followed by surgery [3]. The addition of nCRT to surgery significantly improved 5- and 10-year overall survival (from 33% to 47% and 25% to 38%, respectively) [4], [5]. Perioperative chemotherapy has recently been introduced as an alternative standard for adenocarcinoma [6].

Radiotherapy for esophageal cancer affects surrounding organs-of-interest (OOIs), like the heart and lungs. This may cause acute and late side-effects, increasing the risk of postoperative complications, pulmonary and cardiac events, and immune suppression (i.e. radiation-induced lymphopenia (RIL)) [7], [8], [9], [10], [11]. Improvement in cancer-specific survival leads to rising mortality from non-cancer-related causes [11], [12], making it increasingly important to reduce the risk of long-term treatment sequelae. Higher cardiac dose is associated with major coronary events, arrhythmias, and decreased survival [13], [14], [15], [16], [17], [18], while increased lung dose correlates with pulmonary adverse-events and worse survival outcomes [19], [20], [21], [22], [23], [24]. Additionally, RIL is also linked to higher radiation doses and worse survival outcomes [9], [25], [26], [27], [28], [29]. This further emphasizes the clinical importance of OOI dose reduction in esophageal cancer radiotherapy.

Current clinical practice for esophageal cancer generally involves image-guided radiotherapy (IGRT), combining in-room cone-beam computed tomography (CBCT) with advanced delivery methods like intensity modulated radiotherapy (IMRT) or volumetric modulated arc therapy (VMAT) [3]. IGRT enables daily position verification and repositioning to improve target coverage while reducing radiation exposure to OOIs [30], [31]. However, IGRT for esophageal cancer is limited by poor soft-tissue contrast, making direct tumor localization challenging. As such, setup verification relies on bony anatomy, which does not account for significant tumor motion and deformations, leading to potential compromised target coverage. Consequently, generous planning target volume (PTV) margins are required to ensure clinical target volume (CTV) coverage, increasing radiation-associated side-effects to nearby OOIs.

To address these limitations, Jin et al. investigated fiducial markers to quantify interfractional tumor motion and improve setup verification in esophageal cancer radiotherapy [32]. Our findings confirmed that tumor motion was greatest in the cranial-caudal direction, and that significant deformation occurs, particularly in the proximal stomach. However, marker-based registration proved clinically impractical due to tissue deformation and the complexity of manual target alignment between daily CBCT and planning computed tomography (pCT) scan [32]. Another approach to overcome limited soft-tissue contrast is with MR–guided radiotherapy (MRgRT), which provides superior soft-tissue contrast [33]. However, its limited availability and prolonged treatment times [34], restrict routine clinical use.

Daily online adaptive radiotherapy (oART) using CBCT has emerged as an alternative to the limitations of IGRT and MRgRT. In pelvic and abdominal malignancies, oART-enabled margin reduction has been reported, with planning target volume (PTV) margins decreasing from approximately 10 mm to 2–5 mm, resulting in reduced doses to OOIs while maintaining target coverage [35], [36], [37]. However, comparable data for esophageal cancer are currently lacking.

In oART, deformable image registration of the pCT to the daily CBCT, combined with artificial (AI)-assisted auto-segmentation and (limited) manual edits propagates reliable target and OOI contours despite the low intrinsic soft-tissue contrast of CBCT [38]. This approach mitigates, rather than improves, the CBCT image-quality limitations. By generating a new plan based on daily anatomy, CBCT-based oART compensates for interfractional variations and permits smaller PTV margins, reducing OOI dose while maintaining or improving target coverage [39]. Furthermore, compared to MRgRT, CBCT-based oART offers greater clinical accessibility and shorter treatment sessions. The effectiveness of CBCT-based oART has been demonstrated across multiple malignancies including rectal cancer, bladder cancer, and bone metastases, where CBCT-based oART maintained target coverage while lowering OOI doses [38], [40], [41].

Two recent studies by Bachmann et al. [42], [43] provided the first clinical evaluation of CBCT-based oART for esophageal cancer. The study reported that the treatment plans were adapted in 99% of the fractions, resulting in a reduction in mean heart dose, decrease in V_20Gy_ of the lungs, and improved target coverage [42], [43]. Importantly, the studies did not adapt their clinical IGRT CTV and PTV margins.

The aim of this emulation study in patients with esophageal or GEJ cancer receiving nCRT was to assess potential benefits of daily oART with reduced PTV margins compared to non-adaptive IGRT.

## 2 Materials & methods

### 2.1 Study population

This single-center retrospective cohort study was approved by the institutional review board (2026.0017). Written informed consent was obtained from all patients. Ten consecutive patients who underwent nCRT for locally advanced esophageal (n = 8) or GEJ cancer (n = 2) at Amsterdam UMC between March and September 2023 were included. Included patients had no second primary tumor in the esophagus, completed all prescribed fractions, and received the planned nCRT regimen without escalation to a definitive chemoradiotherapy protocol. During the clinically delivered non-adaptive IGRT course, two patients required a new treatment plan due to anatomical changes. Baseline characteristics are summarized in Table 1.

**Table 1:** Baseline characteristics of the 10 retrospectively included patients with esophageal cancer treated with neoadjuvant chemoradiotherapy.

Characteristic	n
Age, years (mean ± standard deviation)	68.4 ± 8.4
Male sex	9
WHO performance status 0 1	9 1
Clinical T-stage cT2 cT3	1 9
Clinical N-stage cN0 cN1 cN2	3 6 1
Tumor histology Adenocarcinoma Squamous cell carcinoma	9 1
Tumor location Distal third of esophagus Gastroesophageal junction	8 2
New radiotherapy plan during treatment No Yes	8 2

### 2.2 Treatment

Chemotherapy consisted of 5 courses of weekly intravenous carboplatin and paclitaxel with concurrent radiotherapy of 41.4 Gy in 23 fractions, followed by surgery (i.e. the CROSS regimen) [3]. Prior to radiotherapy, each patient underwent a 10-phase four-dimensional CT (4D-CT) scan to create the clinical treatment plan. The average intensity projection (AVE-IP) from the 4D-CT was used as the pCT. A diagnostic ^18^F-FDG positron emission tomography (PET)/CT was registered to the pCT to assist in the delineation of the gross tumor volume (GTV) encompassing the macroscopic primary tumor and involved lymph nodes.

Target delineation followed the institutional protocol derived and slightly modified from the CROSS trial [3]. The gross tumor volume (GTV) encompassed all visible primary tumor and involved lymph nodes on CT, PET, and endoscopy. To create the internal clinical target volume (ICTV), the GTV was expanded by 5 mm in lateral and antero-posterior (AP) directions (adjusted for anatomical borders), 20 mm in craniocaudal (CC) direction, and further expanded to account for all 10 phases of the respiratory cycle (on the 4D-CT). The PTV was generated by expanding the ICTV by 5 mm in the AP direction, 7 mm in the left–right (LR) direction, and 10 mm in the CC direction.

All patients were treated on a Ethos machine following our departmental IGRT protocol using 2 or 3 arc VMAT plan, generated in Eclipse (version 16.1), clinical dose-guidance parameters used to create the plans are detailed in Table S.1. Clinical treatment plans delivered a median of 496 monitor units (range: 349–538). Before each fraction, a 16 s CBCT scan was performed after patient setup. This scan was used to compare the patient’s actual position with the planned positioning, and 3D corrections where made by aligning the bony anatomy. The patient’s setup was then verified to ensure that the ICTV was fully encompassed by the PTV; if this was not the case, the positioning was corrected accordingly. If significant internal anatomical changes prevented accurate delivery, a new treatment plan was generated offline.

### 2.3 Emulating online adaptive radiotherapy

CBCT-based oART was emulated in a test environment (Ethos v1.1, Varian Medical Systems, Palo Alto, CA); step-by-step details are provided in Supplementary Materials section B. In short, firstly as oART mitigates interfraction variation, PTV margins in the emulated plans were reduced to 3 mm (AP) and 5 mm (LR/CC) based on institutional oART experience, while target/OAR goals were unchanged. Using these parameters a new 2–3 arc VMAT treatment plan was generated. Next for each fraction, the routine IGRT CBCT was imported, and a synthetic CT was generated by deformably registering the pCT to the CBCT; contours were propagated with artificial intelligence (AI) assistance, evaluated by the clinician and manually adjusted if deemed necessary, after which an adaptive plan was optimized on the synthetic CT.

### 2.4 Dose volume histograms analysis

In order to compare the dose-volume parameters between the adaptive treatment and the non-adaptive IGRT (as planned and delivered), the corresponding dose volume histograms (DVH) were calculated. To determine the clinically delivered non-adaptive DVHs for each fraction the following steps were followed. First, for each patient a rigid bone-match registration on the spine was performed between the daily CBCTs and the reference CT. The pCT was typically used as reference; however, for patients with a re-optimized treatment plan the updated plan and contours were used for subsequent fractions. Next, using this registration, daily adapted contours (created during the oART workflow) were transferred to the pCT. Finally, the IGRT plan was evaluated using the adapted contours, reflecting the actual position of the OOIs and target on the day of treatment, to determine the clinically delivered DVHs.

The potential benefits of oART compared to the current non-adaptive IGRT workflow, as clinically planned and delivered, were evaluated based on several DVH parameters. All dose–volume metrics except mean dose were evaluated per-fraction against fraction-equivalent (FE) thresholds; mean dose was compared with the clinical cumulative threshold, as it is insensitive to intra-structure dose distribution. FE-thresholds were defined as per-fraction equivalents of the clinical cumulative constraints by dividing each clinical threshold by the number of fractions (n = 23), ensuring that if the FE-threshold is respected at every fraction, the cumulative clinical constraint cannot be exceeded.

DVH parameters of the ICTV and the two major OOIs, heart and lungs, were compared, since these structures are clinically relevant OOIs due to their proximity to the esophagus and susceptibility to anatomical changes. To this end we compared in how many of the delivered fractions the ICTV FE clinical threshold of target coverage (V_95%_ > 98%) and hotspot dose (D_0.1cm_^3^ < 110%) was violated. In accordance with institutional OOI constraints, for the heart mean dose (D_mean_) and FE-V_30Gy_ (V_1.3Gy_) were evaluated, while for the lungs Dmean, FE-V_20Gy_ (V_0.87Gy_) and FE-V_10Gy_ (V_0.43Gy_) were assessed.

Finally, differences in PTV size between the IGRT and oART workflow were assessed, as well as changes in ICTV size measured during the oART workflow over the course of treatment.

### 2.5 Statistical analysis

Baseline patient-, tumor-, and treatment-related characteristics are presented in Table 1. To assess statistical significance between differences in DVH parameters of the planned, non-adapted, and adapted plans, Wilcoxon’s signed-rank tests were used, and results are reported as medians with interquartile range (IQR) and p-values. A linear mixed-effects model was used to evaluate changes in ICTV volume over time, with fraction number as a fixed effect and a random intercept per patient to capture within-subject variability. All statistical analyses were conducted using Python (version 3.12.4). A p-value < 0.05 was considered statistically significant.

## 3 Results

**Fig. 1:**
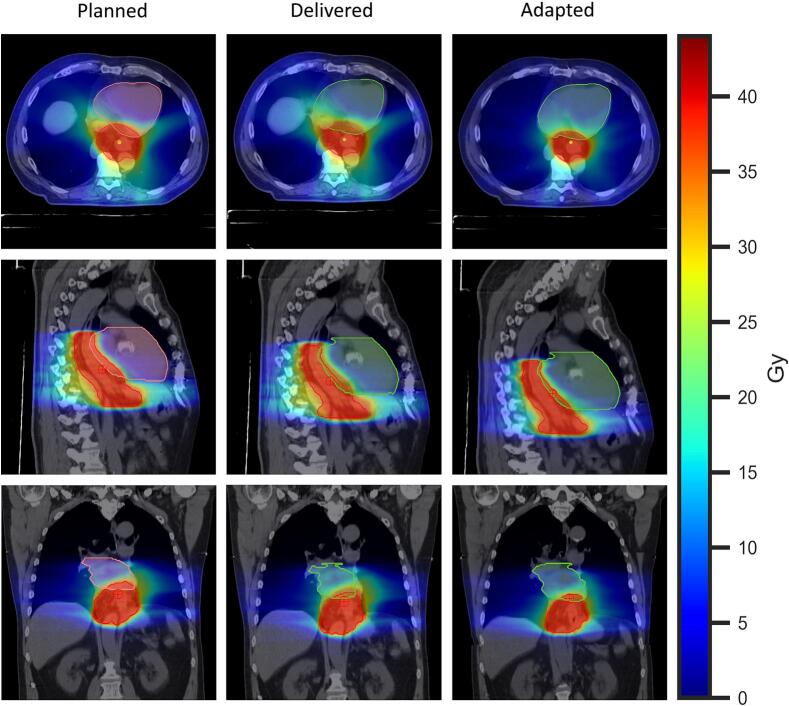
Example of the axial, sagittal and coronal slices from one patient showing the clinically planned, delivered, and the adapted workflows. The Planned column includes the planning CT with IGRT treatment plans and contours; the Delivered column shows a synthetic CT from one fraction with the IGRT plan and adapted contours; and the Adapted column displays the same synthetic CT and contours with the adapted oART plan.

**Fig. 2:**
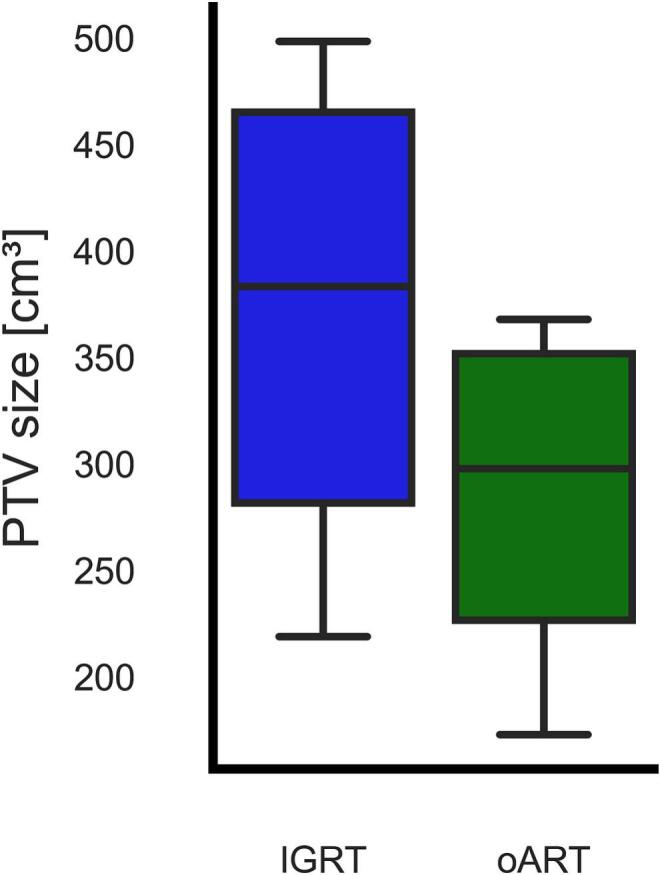
Boxplots showing the reduction in planning target volume (PTV) (cm^3^) between the clinically used IGRT PTVs and the online adaptive radiotherapy (oART) PTVs of the 10 patients. Boxes extends from quartile 1 to 3, the horizontal line represents median value and whiskers extend to 90% of the distribution.

The CBCT image quality was adequate for emulating oART successfully in all 230 fractions as the different structures were sufficiently visible to be delineated by a clinician, an example of the resulting plans and contours are shown in Fig. 1. The implementation of oART and accompanied PTV margin reduction resulted in a reduction of PTV size, with a median decrease of 24.6% (range 19.9%–34.9% (*p* = 0.002; Fig. 2)). Furthermore we found a 5 cm^3^ reduction in median PTV size over the course of treatment from 288 cm^3^ to 283 cm^3^.

**Fig. 3:**
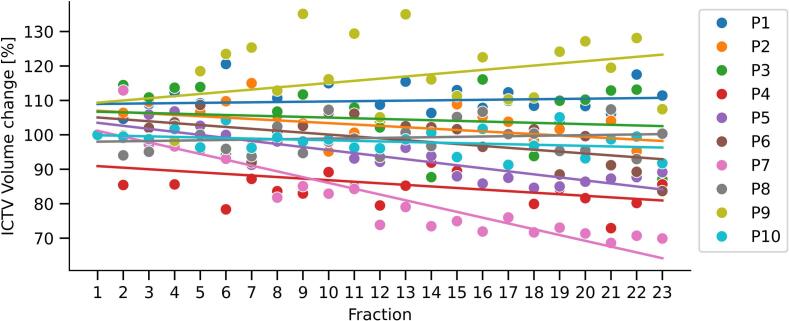
Graph showing the change in ICTV size (%) in oART plans over the course of treatment relative to the ICTV at the start of treatment (fraction 1 = 100%). Lines represent linear fits to the data for each patient (P).

For the ICTV, no significant difference was observed between the median ICTV size measured on daily CBCTs as compared to those from the non-adaptive IGRT pCT (*p* = 0.332). However, a small but significant trend was observed over the course of treatment, showing a mean ICTV size decrease of 0.35% per fraction, corresponding to a mean absolute decrease of 0.75 cm^3^ per fraction (*p* < 0.001; Fig. 3). Furthermore, the daily ICTV volume had a median standard variation of 6.8% (range 3.5%–15.3%).

**Fig. 4:**
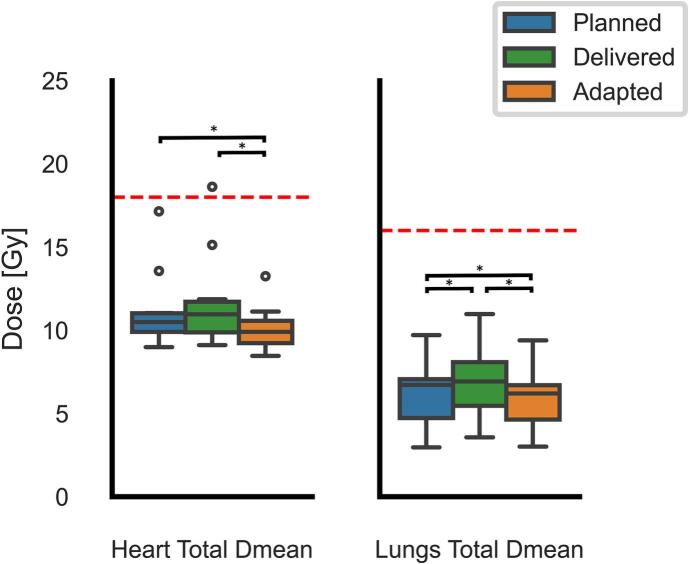
Boxplots showing the cumulative mean heart and mean lung dose (Gy) for the prescribed (blue), delivered (green) and adaptive (orange) plans. The red dotted lines represents the treatment threshold. Boxes extends from quartile 1 to 3, the horizontal line represents median value and whiskers extend to 90% of the distribution. * Denotes statistically significant difference*s* (p < 0.05). (For interpretation of the references to colour in this figure legend, the reader is referred to the web version of this article.)

**Fig. 5:**
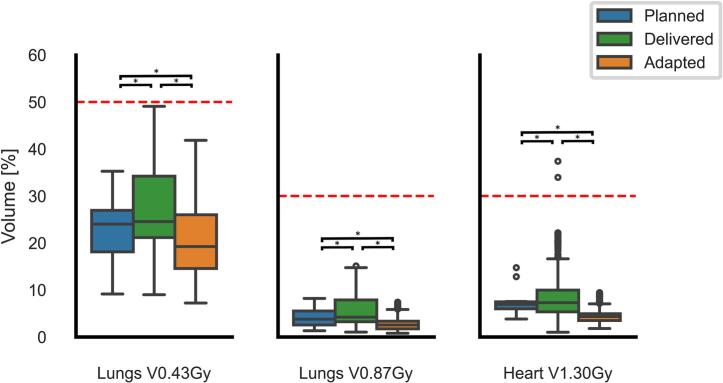
Boxplots showing the relative volume (%) of the fraction-equivalent Heart V_30Gy_ (V_1.30Gy_), Lungs V_20Gy_ (V_0.87Gy_), and Lungs V_10Gy_ (V_0.43Gy_) of each fraction for the planned (blue), delivered (green), and adapted (orange) plans. The red dotted lines represents the treatment threshold. Boxes extends from quartile 1 to 3, the horizontal line represents median value and whiskers extend to 90% of the distribution, dots denote outliers beyond whiskers. * Denotes statistically significant differences (p < 0.05). (For interpretation of the references to colour in this figure legend, the reader is referred to the web version of this article.)

The results of the dose-volume metrics comparison between the planned treatment plans (n = 1 per patient), clinically delivered IGRT (n = 23 per patient), and the adapted (oART) treatment plans (n = 23 per patient) are shown in Fig. 4, Fig. 5. In 13.9% of fractions (n = 32/230), the clinically delivered dose coverage (V_95%_) fell below the 98% planning goal for the ICTV (median = 91.9%, IQR = 89.5%–93.5). This was significantly improved to 0.4% of fractions (n = 1/230; V_95%_ = 97.9%) in the adapted plans (*p* = 0.014). Furthermore, the fractional median hotspot dose D_0.1cc_ within the ICTV was significantly reduced in the adapted plans compared to delivered plans (106.2% vs. 103.9%; *p* = 0.002). PTV V95% dose coverage was similarly improved in the adapted plans compared to the delivered plans (Fig. S.1).

For the heart and lungs, adapted plans consistently outperformed planned and clinically delivered doses. The mean heart dose was reduced by 10% (median [IQR] 11.0[1.8] Gy vs. 9.9[1.3] Gy; *p* = 0.037; Fig. 4), while the heart FE-V_30Gy_ was reduced by 42% (median [IQR] 7.3[4.6]% vs. 4.3[1.4]%; *p* = 0.002; Fig. 5). Similar improvements were observed for the lungs between adaptive and delivered plans, with a 11% reduction in mean lung dose (median [IQR] 6.9[2.6] Gy vs 6.2[2.1] Gy; *p* = 0.002; Fig. 4). Additionally, reduction in lungs FE-V_20Gy_ and FE-V_10Gy_ doses were 39% and 22%, respectively (median [IQR] 4.2[4.6]% vs. 2.6[1.7]%; *p* = 0.002, and median [IQR] 24.6[13.1]% vs. 19.3[11.5]%; *p* = 0.002; Fig. 5).

## 4 Discussion

In this study, we assessed the potential improvements in dose-volume parameters of daily oART with reduced PTV margins, compared to non-adaptive IGRT in patients with esophageal or GEJ cancer. IGRT radiotherapy protocols for esophageal cancer use large PTV margins to compensate for inter- and intrafraction variations, increasing radiation exposure to OOIs and the risk of radiation-associated side-effects. Our study showed that daily CBCT-based oART enables smaller PTV margins, while improving target coverage, even in fractions where standard IGRT with current clinical PTV margins may lead to underdosing. This approach reduced OOIs dose and may reduce treatment-related side effects.

Although our cohort included only distal esophageal and GEJ tumors treated with nCRT, benefits of CBCT-based oART may extend to patients receiving definitive chemoradiation (dCRT). Interfractional variations were comparable across treatment intents, supporting the broad applicability of adaptive workflows beyond the neoadjuvant context. Moreover, the higher total doses in dCRT further increase the value of oART in maintaining target coverage, and limit OOI dose [11], [44], [45]. Interfractional motion is site dependent: distal-GEJ tumors show large shifts driven by diaphragmatic excursion and gastric filling, as such OOI-sparing from oART may be smaller for mid-proximal tumors where these drivers are less pronounced and the heart is typically not a critical OOI [32]. Nonetheless, proximal/mid tumors can exhibit other geometric changes, such as rapid tumor regression during chemoradiotherapy, still warranting for adaptive management.

Previous clinical experience with CBCT-based oART in esophageal cancer, as reported by Bachmann et al., has provided insight into associated dose–volume parameter outcomes [42], [43]. Unlike our study, these maintained standard IGRT CTV and PTV margins in accordance with their institutional practice, resulting in a larger mean PTV size compared to what we report [42]. Both of their studies showed that oART improved target coverage, reduced mean heart dose and lung V_20Gy_ [42], [43]; though the magnitude varied. Their initial study (n = 10) reported reductions of 9.5% and 16.9% in mean heart dose and lung V_20Gy_, respectively [42], whereas their second study (n = 26) observed modest reductions of 3.8% and 3.3% [43]. This difference may be related to increased clinical experience with the adaptive workflow and the larger patient cohort in the second study, leading to more consistent plan adaptation and smaller average OOI dose reductions.

Using reduced PTV margins, our study achieved a 10% reduction in mean heart dose, and a 39% decrease in the lung FE-V_20Gy_. These findings suggest potential for further PTV margin optimization, to improve outcomes and reduce radiation-associated side-effects. Although absolute reductions in mean heart and lung dose were modest, prior studies demonstrated that even small decreases can be clinically relevant: each Gy increase in mean heart dose raised the risk of severe cardiac events and mortality [14], while lower combined heart–lung doses significantly reduced postoperative pneumonia incidence [24].

Our findings regarding target volume reduction align with previous research on esophageal tumors treated with the CROSS regimen [3]. A prior study reported a 28% decrease in mean GTV following treatment, corresponding to a reduction of 0.43 cm^3^ per fraction [46]. Similarly, our study demonstrated an ICTV reduction of 0.75 cm^3^ per fraction, highlighting the impact of GTV shrinkage on ICTV reduction. Furthermore, compared to the prospective study by Bachmann et al., we observed a similar relative PTV reduction over treatment (−6% vs −8%). Unlike conventional IGRT, which does not account for this variability, oART can accommodate these changes by adapting the target contours throughout treatment. However, reducing targets solely based on visible shrinkage risks under-treatment if microscopic disease is not considered, a limitation mitigated in clinical practice through physician oversight.

MRgRT and proton therapy offer advantages for esophageal cancer. Proton therapy markedly reduces cardiac and pulmonary exposure [47]. However, its robustness can be compromised by diaphragmatic baseline shifts and changes in tissue density [48], which may limit performance in highly mobile distal or GEJ tumors. Conversely, MRgRT provides superior soft-tissue contrast and real-time adaptation, though its prolonged treatment sessions, limited availability, and contraindications restrict its routine use [33], [34], [49]. CBCT-based oART is therefore a pragmatic and widely accessible compromise, enabling daily adaptation and clinically meaningful OOI sparing within standard treatment times and at reduced cost compared to MRgRT and proton therapy.

Comparing oART dose-volume benefits across modalities, a preclinical MRgRT study demonstrated considerable mean heart and lung dose reductions (24.3% and 27.8%) [50] compared to our CBCT-based oART study (10% and 11%). In the MRgRT study, CTV-PTV margins were reduced to 2 mm axially and 5 mm craniocaudally to account solely for breathing-related variability, whereas our applied margins (3 mm AP, 5 mm LR and CC) were based on prior institutional adaptive experience and intended to also include setup and delineation uncertainties [50]. The feasibility of such small margins in MRgRT remains uncertain, while previous studies suggest that they adequately account for respiratory motion of esophageal tumors [51], [52], additional uncertainties in delivery accuracy [53] and delineation uncertainty [54], should also be considered when determining CTV-PTV margins. Consequently, clinical oART MRgRT studies adopted larger isotropic margins of 6 mm [34], [55], achieving feasible treatment in patients, with lengthy treatment session times up to 53 min. More recent workflow optimizations have reduced session times in-silico to 23 min while maintaining adequate target coverage in 90% of fractions [55], but feasibility of these shorter treatment session times have not yet been proven in clinical practice. So while MRgRT studies have reported larger OOI dose reductions than our CBCT-based oART results, these potential advantages must be balanced against the substantial treatment times required for MRgRT.

Our study has several limitations. First, for the clinically delivered IGRT dose we assumed a static dose cloud rather than recalculating the original treatment plan on each synthetic CT or CBCT. While this avoids uncertainties associated with dose recalculations [56] of up to 3% error on dose metrics [57], it may not fully reflect anatomical and variations in dose-volume parameters across fractions, such as changes in lung ventilation, gastrointestinal filling, diaphragm position, or patient weight loss. Second, when comparing OOI doses between oART and non-adaptive IGRT, plans were not normalized to identical target coverage. Although normalization could isolate OOI differences independent of coverage, this would not reflect clinical IGRT practice, where actual delivered dose cannot be retrospectively normalized. Third, image registration was performed based on bony anatomy to ensure consistency across fractions and in line with our current clinical practice. While this approach minimizes registration variability, soft-tissue/target-guided matching might yield improved anatomical alignment on CBCT, and thus smaller differences in dose-volume metrics. Next, dose-volume metrics assessments were based on fractional dose distributions rather than accumulated dose. Accurate dose accumulation would require deformable image registration with exceptional precision, which is particularly challenging in homogenous target structures. Additionally, VMAT plans as utilized in this study, are characterized by steep dose gradients, making them highly sensitive to even minor registration errors, which can result in substantial discrepancies in dose-volume metrics [58]. To avoid these limitations of deformable image registration, we reported fractional doses to enable consistent and reliable comparisons. Finally, intrafraction motion was not measured, so margin adequacy is uncertain; the 10-phase 4D-CT ICTV reflects simulation motion only, not day-specific variability. Accordingly, any margin reductions should remain modest until adequacy is promptly validated, ideally through intrafraction motion monitoring or motion-management strategies in future studies.

Finally, although oART can correct for interfractional changes (supporting smaller setup-related PTV margins), the overall PTV size remains limited by intrafraction respiratory motion captured by the ICTV, its ability to reduce the PTV remains limited due to respiratory motion. Currently, Large ICTV margins remain necessary to compensate for respiratory motion [52], [59], and the increased delineation uncertainty caused by motion-induced image artifacts [60]. Integrating respiratory motion management into the oART workflow could help mitigate intrafraction motion, enabling more comprehensive control over both inter- and intrafractional motion during oART, allowing for further margin reductions and improved sparing of OOIs.

In conclusion, this emulation study demonstrated benefits of CBCT-based oART in the treatment of patients with esophageal cancer. This approach effectively accounted for interfraction variability, enabling a reduction in PTV margins. As a result, oART enhanced overall dose-volume outcomes, improved target coverage and significantly reduced radiation exposure to the heart and lungs.

## CRediT authorship contribution statement

**Leigh A.P. Bruijs:** . **Thomas Weststrate:** Writing – review & editing, Writing – original draft, Visualization, Validation, Software, Methodology, Investigation, Formal analysis, Data curation, Conceptualization. **Karin N. Goudschaal:** Writing – review & editing, Data curation, Conceptualization. **Irma W.E.M. van Dijk:** . **Jorrit Visser:** Writing – review & editing, Methodology, Conceptualization. **Joost J.C. Verhoeff:** Writing – review & editing. **Zdenko van Kesteren:** . **Tezontl S. Rosario:** Writing – review & editing, Conceptualization. **Arjan Bel:** Writing – review & editing, Supervision, Resources, Project administration, Funding acquisition, Conceptualization. **Peter S.N. van Rossum:** Writing – review & editing, Supervision, Resources, Project administration, Data curation, Conceptualization.

## Declaration of competing interest

The authors declare the following financial interests/personal relationships which may be considered as potential competing interests: All authors contributed to the present study, which was sponsored by Varian. None of the authors have personal financial or contractual relationships with the sponsor. L.A.P. Bruijs reports that her research position is institutionally funded through a grant from Varian, A Siemens Healthineers Company (Palo Alto, CA, USA), related to the present study. T. Weststrate reports his research position is partially funded by the Dutch Cancer Society (KWF; project 12900). A. Bel reports institutional funding from Varian, for which he has oversight but receives no personal compensation.
