# Supplementary material for: Benefits of daily online plan adaptation with reduced margins in neoadjuvant chemoradiotherapy for esophageal cancer
Source: Phys Imaging Radiat Oncol. 2026 Mar 14;38:100946. doi: 10.1016/j.phro.2026.100946 (PMC13014913; doi:10.1016/j.phro.2026.100946)
Supplement: Supplementary Data 1 [file mmc1.pdf]

Supplementary materials

A. Clinical dose-guidance parameters Table

Table S.1. Structures used for treatment plan creation, including dose parameters and associated target and threshold values.

Structure	dose-parameter	Target value	threshold
PTV	V95%		98%
	Dmax		< 110%
Spinalcord			< 50Gy
Heart	V30Gy	< 12%	< 30%
	Dmean	< 12Gy	< 18Gy
	Dmax		< 104%
Lungs	Dmean	< 7Gy	< 16Gy
	V20Gy	< 4%	< 30%
	V10Gy	< 20%	< 50%
Liver	Dmean		< 26Gy
Kidneys	V18Gy		< 18%
Spleen	Dmean		< 20Gy

B. Ethos emulated adaptive workflow

For the emulated adaptive workflow, previously treated patients with an image-guided radiotherapy (IGRT) baseline plan and corresponding contours were retrospectively reviewed in the test environment under supervision of a radiation oncologist. For each patient, the planning computed-tomography (pCT) scan, associated contours, and the daily IGRT cone-beam CTs (CBCT) acquired during treatment were imported into the Ethos 1.1 emulator. The emulated workflow was as follows:

1. Generating treatment plans: New treatment plans were then created using the IGRT pCT and all original contours—except the PTV, which was reduced to 3 mm in the anterior–posterior direction and 5 mm in the left–right and cranial–caudal directions. These plans were generated using the same planning goals as the original IGRT plans.
2. CBCT import: All daily CBCTs from the IGRT treatment sessions were imported into the emulator one at a time to simulate treatment for each corresponding fraction.
3. Influencer review: The influencers, contours of certain OARs, included the heart and both lungs. These OARs were propagated from the pCT to the daily CBCT using deformable image registration between both image sets. AI assisted in this process by

automatically generating and refining the daily influencer contours, which were then manually adjusted when necessary.

4. Target volume review: The test system propagated the target volumes (GTV and ICTV) from the pCT onto the daily CBCT using the same deformable registration and AI-based contour propagation. When necessary, these target contours were manually adjusted. The PTV was then automatically derived by expanding the ICTV with 3 mm anterior-posteriorly and 5 mm left-right and craniocaudally.
5. Plan adaptation and evaluation: A synthetic CT was generated by combining the Hounsfield Units from the pCT with the anatomy from the daily CBCT. Based on this, a scheduled plan (recalculation of the reference plan) and an adapted plan (optimized on the daily anatomy with the initial constraints) were created.
6. Plan comparison and review: The scheduled and adapted plans were compared in terms of target coverage and OAR doses within the test environment. The adapted plans were reviewed and approved for every fraction.

C. PTV dose-volume parameters

The $V_{95\%}$ of the planning target volume (PTV) was calculated and compared between the delivered image-guided radiotherapy (IGRT) workflow and the adaptive workflow; the results are shown in Figure S.1. In addition, we assessed the number of fractions in which the PTV $V_{95\%}$ fell below the prescribed threshold of 98%. This occurred significantly more often in the delivered workflow (134 of 230 fractions) then the adaptive workflow (2 of 230 fractions).

For the delivered workflow, online adaptive delineations were used to reflect the PTV position on the day of treatment. However, because smaller margins are applied in the adaptive workflow than in IGRT, the number of fractions below the threshold is likely underestimated.

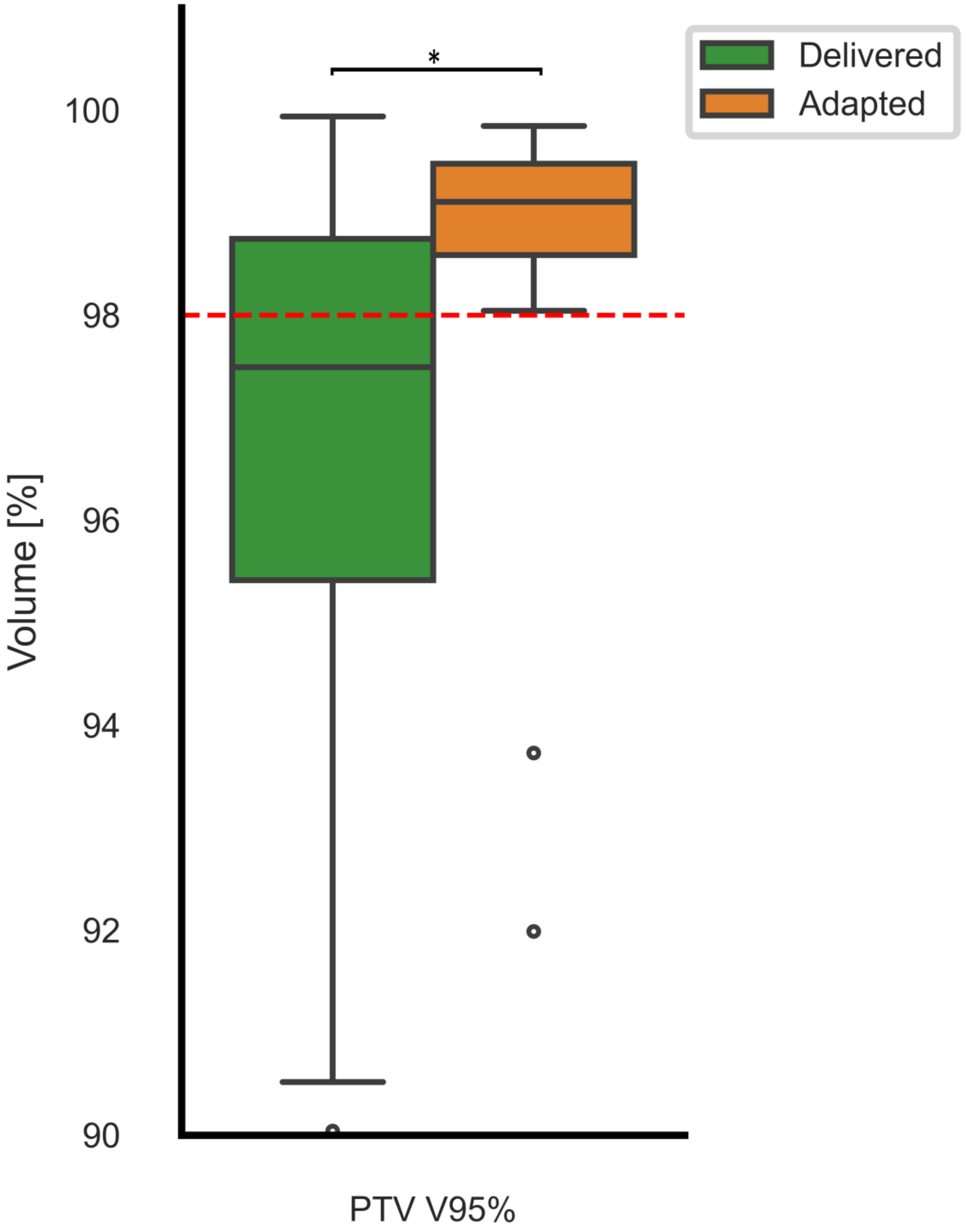

Figure S.1: Boxplots showing the $V_{95\%}$ for the planning target volume (PTV) for each fraction of the delivered (green) and adapted (orange) plans. The red dashed line represents the treatment threshold. Boxes extends from quartile 1 to 3, the horizontal line represents median value and whiskers extend to 90% of the distribution, dots denote outliers beyond whiskers. * Denotes statistically significant differences ($p<0.05$).
